# Clinical Experience with “Stand-Alone” Elephant Trunk Procedure for Descending Aortic Aneurysms

**DOI:** 10.1055/s-0042-1743535

**Published:** 2022-08-07

**Authors:** Ulas Kumbasar, Mohammad A. Zafar, Bulat A. Ziganshin, John A. Elefteriades

**Affiliations:** 1 Aortic Institute at Yale New Haven Hospital, Yale University School of Medicine, New Haven, Connecticut; 2 Department of Cardiovascular and Endovascular Surgery, Kazan State Medical University, Kazan, Russia

**Keywords:** aortic aneurysm, elephant trunk, stand-alone

## Abstract

**Background** Both open and endovascular treatments of descending thoracic aortic aneurysms require a secure proximal landing zone. This may be difficult to achieve when the dilatation extends proximally to the left subclavian level. Clamping above the aneurysm may be difficult. In the case of an endovascular approach, achieving a suitable landing zone may require extensive extra-anatomic debranching, which is not without complications and limitations.

**Methods** We describe a modification of the traditional elephant trunk procedure that represents a “stand-alone” elephant trunk. Under deep hypothermic circulatory arrest, the aorta is transected between the left carotid and left subclavian arteries. A simple, noninverted elephant trunk is placed through the distal cut aorta. The two ends are sewn back together, incorporating the lip of the elephant trunk in the anastomosis. We review our experience in five patients who underwent this procedure.

**Results** All 5 patients (4 males, 1 female) aged 41 to 68 (mean, 57 years) tolerated the Stage 1 stand-alone elephant trunk procedure well, without mortality, stroke, or bleeding. The Stage 2 descending aortic replacements were performed at a mean of 6.7 months after Stage 1. There was no Stage 2 mortality, stroke, or bleeding. One patient died 8 years later of cardiac cause, and the remaining are alive and well.

**Conclusion** A stand-alone elephant trunk procedure is safe and straightforward and provides an excellent proximal foundation for subsequent open (or potentially endovascular) descending aortic replacement.

## Introduction

Patients with aortic disease involving the descending thoracic aorta present a major surgical challenge due to the extent of the disease and the morbidity and mortality associated with the repair procedure. 1 Depending on the extent/anatomy of the disease they may require more than one surgical intervention. Although endovascular repair techniques are associated with less morbidity compared with open repairs, they may not be technically suitable for all patients and their durability is uncertain. 2

Repair of descending aortic disease (open or endovascular) requires a securely landed graft at the distal aortic arch level. In the present era, this is often accomplished by cumbersome extra-anatomic debranching of the aortic arch. We report our experience with an alternative procedure, the “stand-alone” elephant trunk (ET). For this procedure, we transect the aortic arch between the left carotid and left subclavian arteries. We then sew the two sides together, incorporating a Dacron ET in the distal segment. We performed this procedure in 5 patients between January 2010 and June 2020 and analyzed our results.

## Methods

We reviewed our institution's (Aortic Institute at Yale-New Haven) experience with 5 patients who underwent “stand-alone” ET procedure by retrospective chart review. This study was approved by the Yale University Human Investigations Committee, and the requirement for individual consent was waived. The Yale Aortic Institute database was searched for the period from January 1, 2010 to July 30, 2020. Each patient's data were collected including the following variables: sex, age, the occurrence of stroke or paraplegia, need for surgical reexploration for each stage, period between two stages, follow-up details, and overall mortality. Patients were followed up through June 2020.

### Surgical Technique

#### Stage 1



**Video 1** Surgical video illustration of the stand-alone elephant trunk procedure. [Video courtesy: Elefteriades JA, Ziganshin BA. Practical Tips in Aortic Surgery. Springer; 2021.]


**Fig. 1:**
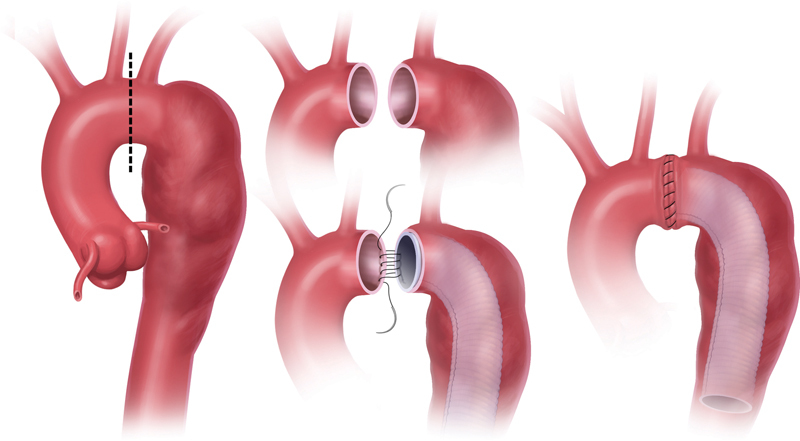
Establishment of a stand-alone elephant trunk.

Stage 1 is performed through a standard median sternotomy. Standard cardiopulmonary bypass (CPB) is instituted via the left femoral artery and right atrium. Straight deep hypothermic circulatory arrest (DHCA) is utilized during the ET placement. The head is packed with ice. We do not use cerebral perfusion techniques, either antegrade or retrograde. Following exposure of the arch vessels, the distal ascending aorta is cross-clamped and the first dose of cardioplegia is given. Once the nasopharyngeal temperature reaches 18 to 20°C, the patient is put in the Trendelenburg position, circulatory arrest is instituted, and the cross-clamp is removed. We transect the aortic arch between the left carotid and left subclavian arteries (perpendicular to its long axis). We then sew the two sides of the aorta together, incorporating a Dacron ET in the distal segment ( Fig. 1 and Video 1 ). After completion of the anastomosis, the aorta is de-aired, DHCA is terminated, CPB flow is increased gradually, and rewarming begun. When the bladder temperature reaches 34°C, the patient is weaned from CPB.

In terms of length of the ET, we make certain that it is long enough to reach well beyond the subclavian artery, so that it can be retrieved easily at Stage 2. The paraplegia risk from the ET before Stage 2 is minimal for two reasons: First, the aorta in which the ET lies is quite dilated (that is the reason for the procedure in the first place, so there is little danger of the free-floating graft occluding vital intercostal arteries). Second, unlike a stented graft, the free-floating ET does not lie against the spinal ostea with radial force.

Because the incision and anastomosis are done proximal to the left subclavian artery, the recurrent laryngeal nerve is not in jeopardy.

#### Stage 2

**Fig. 2:**
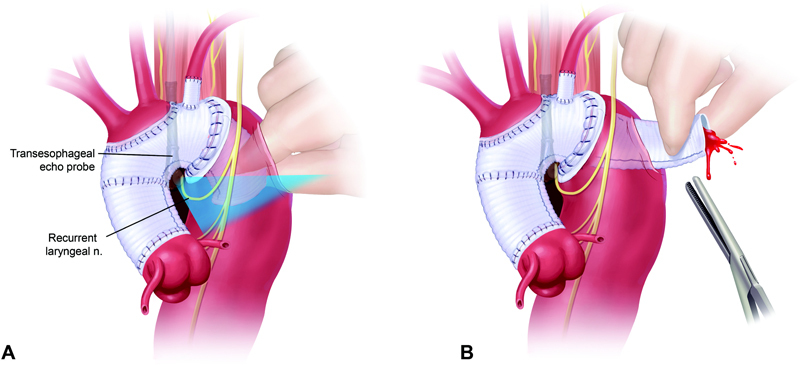
( **A** ) Illustrated is the transesophageal echocardiography-guided approach to the elephant trunk graft. Please note the location of the recurrent laryngeal nerve, which is protected by this technique. ( **B** ) Schematic illustration of elephant trunk graft retrieval in preparation for clamping. Image courtesy: Ziganshin and Elefteriades. 3

General anesthesia is induced with a double-lumen endotracheal tube, cerebral spinal fluid drainage is used to optimize spinal cord protection, the patient is positioned in the right lateral decubitus position, and a left thoracotomy incision is made. Cannulation is performed via the left inferior pulmonary vein and femoral artery. The ET graft in the descending aorta is visualized by transesophageal echocardiography. The ET is retrieved by the “finger-thumb technique” 3 ( Fig. 2 ). Adenosine is administered for cardiac standstill (usually 16 g bolus) and a small vertical aortotomy is done in the descending aorta (to create a tight fit for the surgeon's finger and thumb) at the level of the ET graft. The graft is retrieved by the finger-thumb technique, brought out of the aorta under finger-thumb control, and clamped. (We find that the 15–20 seconds of asystole induced by the adenosine is generally adequate to permit opening the aorta to retrieve and clamp the ET graft.) The length of the ET is adjusted by pulling the graft distally (to undo interim retraction and to spread the corrugations), and the distal anastomosis is performed. This anastomosis is done to the transected distal aorta below the lower extent of the aneurysm. If needed, an identical piece of Dacron graft is anastomosed to the original graft to provided additional length. Left heart bypass is terminated and the remaining aortic tissue is wrapped around the anastomosed graft. Intercostal arteries are managed by our usual routine for descending aortic replacement. (Preoperative computed tomography angiography of the spinal arteries is performed. Any identified spinal artery is spared or reimplanted at the time of surgery. 4 ) At the end of the surgical procedure, the left subclavian artery is transected and connected to blood flow via a separate 8- or 10-mm Dacron graft originating from the new aortic graft or the native aorta.

## Results

There were 5 patients (4 males, 1 female) aged 41 to 68 (mean, 57 years). All procedures were elective. We used DHCA for all cases. There was no operative mortality, stroke, or need for reexploration due to bleeding in the first stage. All patients survived to have the second stage procedure. The mean period between the stages was 6.7 months. The second stage was performed open for all patients. There was no mortality, stroke, or paraplegia in the second stage, either. The mean postoperative follow-up period was 70.2 months overall. Four out of 5 (80%) patients were alive at follow-up. One patient died due to sudden cardiac arrest of unknown etiology 8 years after the second stage procedure.

## Discussion

After the original ET technique, which was described by Borst et al in 1983, the procedure was first modified by Crawford and Svensson. 5 6 7 Variations of this procedure continued to evolve over the years, including reversed ET, bidirectional ET, and more recently the frozen ET technique. 8 9 10 In this report, we introduce another modification of this technique, the “stand-alone” ET procedure, for patients who have descending thoracic aortic disease without ascending and/or aortic arch involvement, and we review our clinical experience.

This procedure is useful and intended for ( 1 ) patients who require open replacement of the descending aorta who have no suitable site for a proximal cross clamp, and ( 2 ) patients who are to undergo endovascular therapy of the descending aorta who require a stable, secure, and durable proximal landing zone.

### Traditional Elephant Trunk

Briefly, the basic principle of the traditional ET procedure includes replacement of the aortic arch coupled with a free-ﬂoating extension of the arch prosthesis which is left behind in the proximal descending aorta in the first stage; in the second stage an extension of the prosthetic graft up is added, replacing the descending aorta to the required level via an open lateral thoracotomy or percutaneously with an endovascular graft in the second stage. 5 7 11 12 The main advantages of this two-staged technique become clearer during the second operation. First, the proximal descending aorta does not need to be dissected, thereby decreasing the risk of injury to the adjacent structures such as the pulmonary artery, esophagus, lymphatic structures, and recurrent laryngeal nerve. Second, once the ET is accessed and clamped under echo guidance a graft-to-graft extension can be added with great rapidity, thus minimizing cross-clamp time and duration of spinal ischemia. Third, this technique avoids the need for DHCA during the second stage. Also, with this procedure the need to clamp the proximal left subclavian artery may be avoided, thereby the risk of stroke and paraplegia is reduced. 11 13

### “Stand-Alone” Elephant Trunk

This modification is intended for the subset of patients who have severe descending thoracic disease without ascending and/or aortic arch involvement. The “stand-alone” ET technique provides a “rock stable” securely landed graft at the aortic arch level without any need for an extra-anatomic debranching of the aortic arch level. This greatly facilitates the second stage open procedure for descending aortic replacement. In terms of potential endovascular therapies (for those teams that prefer such an approach), this stand-alone technique provides an alternative to debranching thoracic endovascular aortic repairs (d-TEVAR procedures).

We performed this procedure in 5 patients. There was no early mortality, no stroke, no paraplegia, and no need for reexploration due to bleeding in either stage.

### Perspective

This stand-alone ET procedure represents another alternative in a large armamentarium of options for the aneurysmal descending aorta. This is intended specifically for the subset of patients in whom the aortic arch is normal in size and shape. When the arch itself is dilated, a traditional arch replacement and standard ET are appropriate, with a later second stage via thoracotomy. It is also important to keep in mind alternative one-stage procedures that can accomplish arch and descending aortic replacement in one sitting: namely, arch and descending aortic replacement under DHCA with open proximal anastomosis via left thoracotomy, or arch and descending aortic replacement via mid-sternotomy supplemented by T-left thoracotomy, or bilateral anterior thoracotomy (clamshell). Also, an endovascular approach by covered stent graft (after any requisite debranching via sternotomy) is another popular alternative. We feel the stand-alone ET procedure described here offers the powerful advantage of a secure, solid proximal anastomosis.

In conclusion, even though this experience reflects the results of a limited number of patients in a single institution, the “stand-alone” ET procedure is a unique modification that can be performed in a subset of patients with descending thoracic aortic disease without ascending and/or aortic arch involvement, providing technical advantages with low major morbidity and mortality.
